# Opinion: PARP inhibitors in cancer—what do we still need to know?

**DOI:** 10.1098/rsob.220118

**Published:** 2022-07-27

**Authors:** Andrew J. Wicks, Dragomir B. Krastev, Stephen J. Pettitt, Andrew N. J. Tutt, Christopher J. Lord

**Affiliations:** ^1^ The CRUK Gene Function Laboratory, The Institute of Cancer Research, London SW3 6JB, UK; ^2^ Breast Cancer Now Toby Robins Breast Cancer Research Centre, The Institute of Cancer Research, London SW3 6JB, UK

**Keywords:** PARP inhibitors, cancer, synthetic lethality, biomarkers, drug resistance

## Abstract

PARP inhibitors (PARPi) have been demonstrated to exhibit profound anti-tumour activity in individuals whose cancers have a defect in the homologous recombination DNA repair pathway. Here, we describe the current consensus as to how PARPi work and how drug resistance to these agents emerges. We discuss the need to refine the current repertoire of clinical-grade companion biomarkers to be used with PARPi, so that patient stratification can be improved, the early emergence of drug resistance can be detected and dose-limiting toxicity can be predicted. We also highlight current thoughts about how PARPi resistance might be treated.

## Graphical abstract

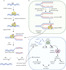

## 1. What do we know?

### 1.1. PARP1 function

The target of clinical PARPi, PARP1 (poly[ADP-ribose] polymerase 1), is classically known for its role as a sensor of DNA damage and mediator of DNA repair. PARP1 mediates these effects via its ability to synthesise branched poly(ADP-ribose) (PAR) chains on substrate proteins (PARylation) and also itself (autoPARylation) [1,2]. For example, PARP1 plays a major role in promoting the repair of single-strand DNA breaks (SSB), including unligated Okazaki fragments that escape processing by the DNA repair enzymes FEN1 and LIG1 [3,4]. As part of its repair functions, PARP1 binds to damaged DNA, including SSBs, via N-terminal zinc-finger (ZnF) domains [5,6]. DNA binding invokes a conformational change in PARP1 that causes the release of an autoinhibitory interaction between the helical domain (HD) and the catalytic ADP-ribosyl transferase domain (ART); this, in turn, allows the PARP1 cofactor, NAD^+^ to access ART. The subsequent PARylation of substrate proteins involved in DNA repair enables their retention at the site of DNA damage, the relaxation of chromatin structure to increase access for DNA repair machinery and the repair of damaged DNA [7]. For example, PARylation leads to the recruitment of XRCC1, which in turn leads to the XRCC1-mediated recruitment of single-strand break repair (SSBR) proteins including DNA ligase 3 (LIG3) and DNA polymerase *β* (Pol*β*) [8,9]. PARP1 also autoPARylates, an event that drives its dissociation from DNA [1,2,10]. The PARylation status of PARP1 is also controlled by PAR glycohydrolase (PARG) and ARH3 whose activity enhances the retention of PARP1 on DNA [11,12].

PARP1 is also activated upon binding to double-strand DNA breaks (DSBs) and plays a role in the rapid recruitment and activation of the DNA-damage-sensing MRE11 and NBS1 components of the MRN (MRE11-RAD50-NBS1) complex to DSBs; the MRN complex generates 3′ single-stranded DNA (ssDNA) overhangs required for homologous recombination (HR) [13]. The activity of Ataxia telangiectasia mutated (ATM), a major activator of DSB repair pathways, is also controlled by an interaction with PAR chains [14,15]. In addition, the BRCT domain of BRCA1 recognises PAR chains and PARP1 activity plays an important role in the recruitment of BRCA1 to DSBs [16]. Finally, PARP1 has also been implicated in the suppression of DSB repair by non-homologous end joining (c-NHEJ); PARP1 PARylates the Ku70/80 NHEJ complex, decreasing its affinity for DNA [17]. Additionally, by competing with the Ku70/80 complex for access to the DNA ends, PARP1 may also act to suppress c-NHEJ and promote alternative NHEJ (alt-NHEJ, also known as microhomology end joining or theta-mediated end joining) [18].

### 1.2. PARP1 inhibitors and cancer synthetic lethality

**Figure 1.**
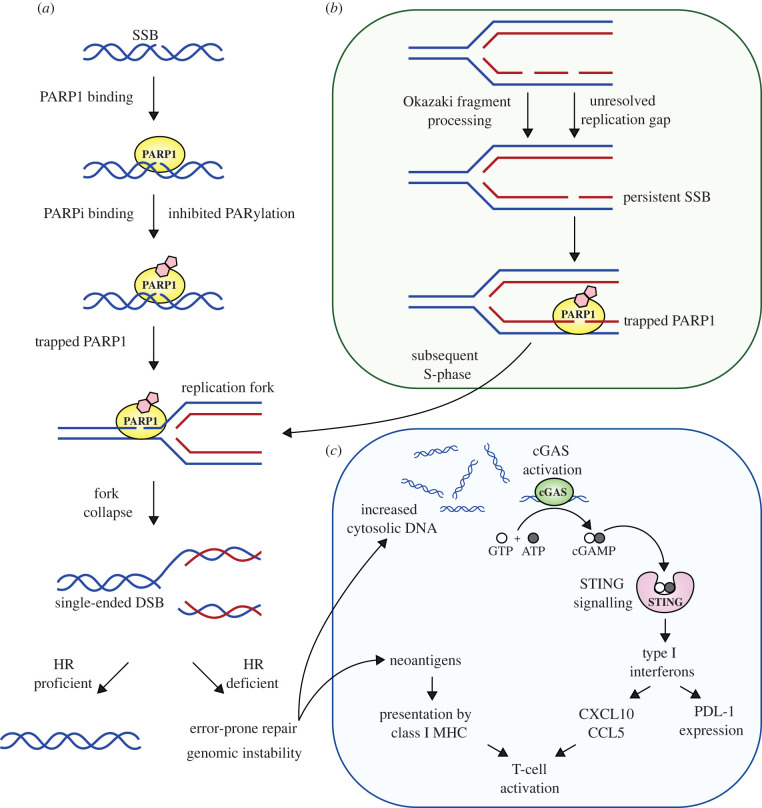
Mechanisms of anti-tumour activity of PARPi. (*a*) PARP1 recognizes DNA lesions, such as single-stranded breaks (SSBs). DNA binding induces conformational changes in PARP1, including a change in an autoinhibitory interaction between the helical (HD) and catalytic (ART) domains; this in turn enables NAD+ to access the catalytic site where it initiates PARylation. PARPi bind the ART domain and inhibit catalytic activity but also alter the conformation of PARP1, trapping PARP1 at the site of DNA damage. Trapped PARP1 forms a replication barrier, leading to fork stalling and collapse. When there is a homologous recombination (HR) defect, error-prone DNA repair pathways are used to repair and restart the replication fork, events that can lead to increased genomic instability and loss of fitness. (*b*) Recent work suggests that unligated Okazaki fragments can form persistent SSBs that are bound by PARP1. In addition, defects in BRCA1/2 also lead to an increase in post-replicative SSBs. When PARP1 is trapped at post-replicative SSBs, this eventually poses an obstacle for the replication fork during the subsequent S phase. (*c*) The DNA damage induced by PARPi causes the generation of cytosolic DNA, which activates the cyclic GMP-AMP (cGAMP) synthetase (cGAS) DNA sensor. This in turn activates stimulator of interferon genes (STING) signalling and the production of type-I interferons and pro-inflammatory chemokines (e.g. CXCL10, CCL5) which, alongside the presentation of neoantigens created upon genomic instability, results in the activation of CD4^+^ and CD8^+^ T cells.

Figure 1 illustrates the mechanisms of anti-tumour activity of PARPi. Existing clinical PARP1 inhibitors bind the catalytic domain of PARP1 and prevent PARylation by structurally mimicking nicotinamide, the by-product of the PARylation reaction [19–21]. In addition to preventing PARylation, clinically-approved PARPi (such as olaparib, niraparib, rucaparib and talazoparib) extend the retention of PARP1 at the site of DNA damage (PARP1 trapping), an effect probably caused by PARPi inducing a conformational change in the structure of PARP1 that increases the avidity of the ZnF for DNA [22–24]. Similar to other ‘trapped’ DNA-associated proteins, trapped PARP1, can be removed from DNA by the p97 segregase [25]. The importance of PARP1 trapping in PARPi-induced cytotoxicity is perhaps best illustrated by the observation that genetic deletion of *PARP1* or mutation of PARP1 ZnF domains causes profound PARPi resistance [23,26,27].

One model to explain the cytotoxicity caused by PARPi suggests that PARP1 becomes trapped by PARPi at unligated Okazaki fragments, with the result that cells undergo mitosis with persistent SSBs and trapped PARP1 [3]. In the subsequent S phase, trapped PARP1 forms a replication barrier, leading to fork stalling and collapse, an event which normally requires homologous recombination (HR) for successful repair [28]. HR is controlled by a series of tumour suppressor proteins including BRCA1, BRCA2, PALB2, RAD51C and RAD51D. A further iteration of this mechanistic model suggests that defects in BRCA1/2 themselves (and their associated DNA recombinase, RAD51) cause an accumulation of post-replicative ssDNA gaps; when combined with the increase in post-replicative ssDNA gaps caused by PARPi, the RPA exhaustion that ensues causes cell death [29,30]. In addition, other DNA lesions enhance PARPi sensitivity, such as those caused by the processing of genomic uracil [31] or DNA alkylation [32]. These DNA lesions are processed to form PARP1 binding sites, which in the presence of PARPi, cause increased PARP1 trapping and enhanced PARPi sensitivity [24].

Recent work has also highlighted a contribution of the immune system to the antitumour efficacy of PARPi. For example, the DNA damage that PARPi elicit has been shown to cause the generation of cytosolic DNA, which in turn is recognized by the cyclic GMP-AMP (cGAMP) synthetase (cGAS) DNA sensor; cGAS recognition of cytosolic DNA activates stimulator of interferon genes (STING) signalling, type-I interferon and pro-inflammatory chemokine production and CD4^+^ and CD8^+^ T cells [33–37]. Importantly, the anti-tumour activity of PARPi in tumour-bearing mice is impaired by CD8^+^ T-cell depletion, or by neutralization with an anti-CD8 antibody [34,35], suggesting that the adaptive immune system also plays a role in PARPi efficacy.

The synthetic lethality between *BRCA1*, *BRCA2* and PARPi seen in pre-clinical models [38,39], also extends to clinical synthetic lethality [40], with PARPi now forming part of the standard-of-care approaches for the treatment of breast, ovarian, prostate or pancreatic cancers with defects in DNA repair by homologous recombination [41]. For example, in gynaecological cancers, the PARPi olaparib is approved for use according to four criteria: (i) as a maintenance treatment for patients with deleterious or suspected germline or somatic *BRCA1/2*-mutated advanced cancer patients who are in complete or partial response to first-line platinum-based chemotherapy (a clinical indication that HR is defective); (ii) as a combination maintenance treatment used in combination with the VEGF inhibitor bevacizumab, in those with HR defective cancer who are in complete or partial response to first-line platinum-based chemotherapy, where homologous recombination deficiency (HRD) is defined by either a deleterious or suspected deleterious *BRCA1/2* mutation and/or an FDA-approved diagnostic that estimates the presence of cancer-associated genomic rearrangements normally associated with HRD; (iii) for the maintenance treatment of patients who are in complete or partial response to platinum-based chemotherapy and (iv) for the treatment of adult patients with deleterious or suspected deleterious germline *BRCA1/2*-mutated (gBRCAm) advanced ovarian cancer who have been treated with three or more prior lines of chemotherapy [42]. The use of olaparib is slightly distinct in breast, prostate and pancreatic cancers, but still focuses on patients who have HR defective cancers, defined either by the presence of deleterious *BRCA1* or *BRCA2* mutations or in the case of prostate cancers, by the presence of deleterious mutations in any one of a panel of genes that control HR [42]. For example, the recently reported OlympiA phase III trial demonstrated the utility of olaparib when used as an adjuvant treatment following standard-of-care chemotherapy in women with *BRCA1* or *BRCA2* mutant, HER2-negative, early breast cancer [43]. Additional PARPi (talazoparib (Pfizer), rucaparib (Clovis Oncology), niraparib (GlaxoSmithKline) and pamiparib (BeiGene) [44]) are also approved for the treatment of cancer, while others (e.g. AZD5305 (AstraZeneca) [45]) are still in clinical development. Future improvements to PARPi could include increased PARP1 specificity to circumvent off-target toxicity [45], decreased PgP efflux to decrease likelihood of this resistance mechanism [46] (see below) and modifications that increase tumour cell-selective PARP1 trapping [22].

### 1.3. PARP1 inhibitor resistance

Despite PARPi being able to elicit significant and sustained anti-tumour responses in some patients, PARPi resistance is a growing clinical problem, particularly in patients with advanced disease [41]. For example, in the Study 10 trial (NCT01482715 – Rucaparib in Patients With gBRCA Mutation Ovarian Cancer), 59.5% of germline *BRCA1/2*-mutant high grade ovarian cancer patients achieved an investigator-assessed confirmed RECIST response to rucaparib, while 40.5% of patients exhibited *de novo* resistance [47]. As well as *de novo* resistance (e.g. no detectable clinical response to PARPi in patients expected to respond due to the presence of a *BRCA1/2* mutation), acquired PARPi resistance is also an issue. In some patients, the cause of PARPi resistance is reversion mutation in either *BRCA1, BRCA2, RAD51C, RAD51D* or *PALB2* [48–54]. These reversion mutations, originally identified in patients with platinum-salt resistance [55,56], are secondary, additional, mutations (i.e. mutations other than the original pathogenic mutation in the gene) that restore the normal open reading frame of the tumour suppressor gene and encode somewhat functional proteins that are able to repair the DNA damage caused by PARPi [48]. Pre-clinical studies have also identified other candidate mechanisms of PARPi resistance. For example, in the absence of BRCA1, HR functionality can be restored by the further loss of DNA end resection inhibitors (e.g. 53BP1, REV7, Shieldin), enabling the resection of DNA ends necessary to initiate RAD51 recruitment [46,57,58]. BRCA1 and BRCA2 also play important roles in protecting stalled DNA replication forks and in the absence of their function, stalled forks are extensively degraded by nucleases such as MRE11 and MUS81, leading to fork collapse [59,60]. PARPi resistance can also be caused by inhibited recruitment of MRE11 and MUS81 [61,62], or increased fork stability via FANCD2 overexpression [63,64].

PARP inhibitor resistance can also occur via upregulation of ABC-family drug efflux pumps that reduce the cellular concentration of PARPi [65]. Although originally identified in a mouse model of BRCA1-mutant cancer [65], *ABCB1* gene fusions that enhance activity have been observed in treatment-refractory breast and ovarian cancers, implying this could also be a cause of clinical PARPi resistance [66,67].

## 2. What don't we know?

Both pre-clinical and clinical investigation have taught us much about how PARPi work and where they might be best used. However, there are still a number of issues that if addressed, could further refine the clinical use of this class of drugs. Two of the key issues, which focus on biomarkers and how PARPi resistance might be targeted, are discussed below.

## 3. Which biomarkers are required to refine the best use of PARPi?

**Figure 2.**
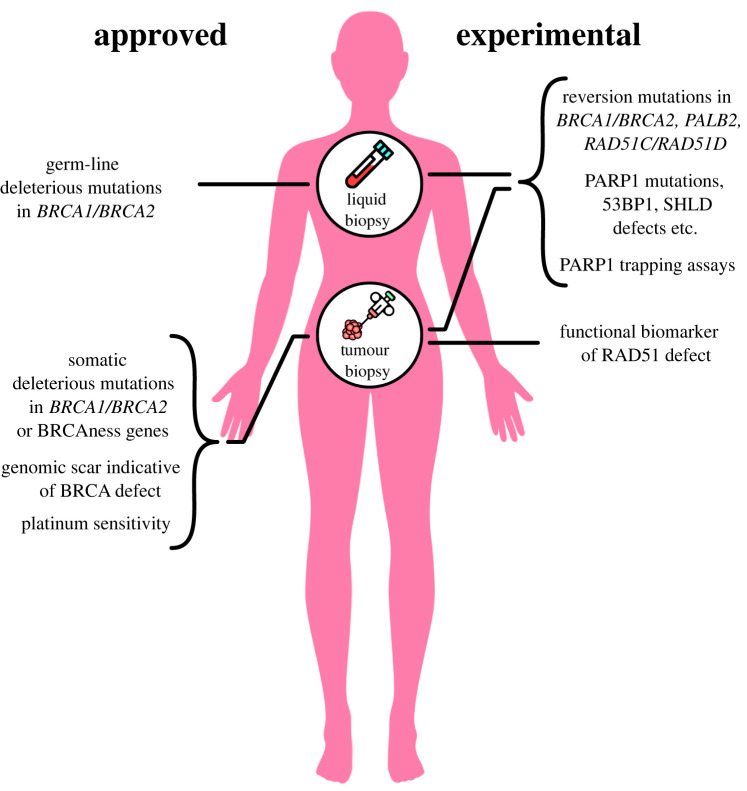
Approved and experimental biomarkers for use with PARPi. Currently approved PARPi companion biomarkers primarily detect either (i) germ-line or somatic mutations in BRCA1/2 or other homologous recombination pathway ‘BRCAness’ genes, or (ii) the presence of HRD-associated genomic scars. Prior platinum salt sensitivity in gynaecological cancers is also used to select patients for subsequent PARPi treatment. Experimental biomarkers that could refine how PARPi are used clinically include the detection of PARPi resistance-causing reversion mutations in BRCA1, BRCA2, PALB2, RAD51C, RAD51D, mutations in PARP1 that impair PARP1 trapping and mutations or reduced expression in genes such as 53BP1, REV7, SHLD1/2/3 that restore HR in BRCA1 mutant tumour cells. These could be used alongside existing biomarkers and also a functional biomarker of the RAD51 defect that characterises a HR defect. There are currently no biomarkers used to predict dose-limiting toxicity and, given the association between PARP1 trapping and the haematological toxicities of PARPi, assays to measure the extent of PARP1 trapping may predict the magnitude of dose-limiting toxicity before it occurs. Figure modified from Lord & Ashworth [41].

At present the biomarkers used to direct the use of PARPi focus on the presence of a deleterious *BRCA1* or *BRCA2* mutation, a deleterious mutation in other tumour suppressor genes that have been implicated in homologous recombination [68–70], prior platinum sensitivity or the presence in the tumour DNA of a genomic scar of HRD. While these biomarkers have utility, there is a clear need to refine the full package of biomarkers that are used to direct the use of this class of drugs (figure 2). We highlight five areas where additional biomarkers could be of use.

### 3.1. Making the distinction between different *BRCA1* and *BRCA2* mutations and different HR-associated genes

At present, the presence of any deleterious mutation in either *BRCA1* or *BRCA2* is sufficient to select a patient for PARPi therapy. Already there is pre-clinical evidence to suggest some pathogenic *BRCA1* mutations are hypomorphs and cause less cellular PARPi sensitivity than others [71–74], although it is not yet known whether the distinctive effect of different *BRCA1* or *BRCA2* mutations extends to distinct clinical responses. Furthermore, there is also the suggestion that some pathogenic *BRCA1* or *BRCA2* mutations are less likely to revert than others (particularly those in splice sites or those that are pathogenic missense mutations [26]). This implies that giving all deleterious *BRCA1/2* mutations equal weight in terms of predicting response and resistance might be short-sighted. Further work (both clinical and pre-clinical) is clearly required to clarify the relative impact each has on PARPi sensitivity and the possibility of resistance. Likewise, although the concept of BRCAness (cancers that phenocopy cancers with *BRCA1/2* mutations [68,75,76]) has proven useful in extending the use of PARPi beyond those individuals with *BRCA1* or *BRCA2* mutant cancers to those with a homologous recombination defect caused by some other means (e.g. mutation in *PALB2, RAD51C, RAD51D*), there is the implicit assumption that each of these other defects causes a similar extent of PARPi sensitivity as for a deleterious *BRCA1* or *BRCA2* mutation. Understanding whether this is the case or not might also allow a refinement of the effectiveness of biomarkers used to direct the use of PARPi.

### 3.2. Clinical-grade assays for identifying reversion mutations

There is already evidence from the study of gynaecological cancers that reversion mutations that are selected for by platinum treatment predict a poorer subsequent response to PARPi [50] and therefore understanding the presence of reversions prior to PARPi treatment is important. Reversions have been detected in circulating tumour DNA from individuals with clinical PARPi resistance [53,54,77,78]; if shown to be present prior to the emergence of clinical resistance (e.g. prior to the detection of a treatment-refractory lesion), detecting reversions in ctDNA could be used to adapt therapy so that resistant tumour cell clones could be targeted before they start to dominate the tumour cell population. We therefore see the development of clinical-grade tests that assay reversions as being critical. These could build on existing DNA sequencing-based assays already applied in the retrospective analysis of clinical trials involving platinum salts or PARPi [48–50,79].

### 3.3. Biomarkers that detect non-reversion mechanisms of PARPi resistance

Although tumour-associated reversions are associated with many cases of PARPi resistance, these are not detected in all cases. Other mechanisms of PARPi resistance have been identified from pre-clinical studies (as detailed above) but as yet, there is only anecdotal evidence for their existence in the clinical disease [26,80]. As such, robust, clinical grade biomarkers that allow these candidate resistance mechanisms to be detected are required. Ideally functional biomarkers (e.g. of 53BP1-Shieldin pathway function or PARP1 trapping) will also be available to interrogate non-mutational loss of these pathways.

### 3.4. Biomarkers of HR function

With the exception of prior platinum sensitivity, all of the existing clinically approved biomarkers used to stratify patients for PARPi treatment seek to identify that there has been an HR defect at some point in the history of cancer. What these biomarkers do not indicate is that the HR defect is present at the time of treatment. For example, HR defects arising via promoter methylation of HR genes (e.g. of *BRCA1*) may be reversed during the course of the disease. Such tumours retain the historical mutational signature of HRD, but have restored HR function and thus are not sensitive to PARP or platinum treatment [81–84]. Efforts are being made to convert a research-used assay of functional HRD, nuclear RAD51, into a clinical grade test which could estimate HR function [85,86]. It seems reasonable to think that such a biomarker might not be used in isolation but would be most powerful when used as part of an algorithm that also includes information from the other aforementioned biomarkers.

### 3.5. Biomarkers that predict dose-limiting toxicity

At present, biomarkers that predict dose-limiting toxicity do not exist. It seems possible that PARP1 trapping in myeloid cells explains (at least in part) the haematological toxicities seen with PARPi [45,87]. Given this, biomarkers that allow PARP1 trapping to be measured in patient samples might be of use and could predict those more likely to eventually show dose limiting toxicity before it occurs (e.g. those with elevated PARP1 trapping in lymphocytes).

## 4. How can we prevent or delay PARPi resistance?

At present, the treatment options for patients who develop PARP inhibitor resistance are limited and tend not to involve a targeted approach based upon the particular molecular make-up of resistant disease. Furthermore, approaches that are proven to delay the emergence of resistance do not exist. Here we highlight three areas of research that could inform how PARPi resistance is managed clinically.

### 4.1. Targeting PARPi resistance when caused by reversion

The study of reversions has suggested that some of the new DNA sequences formed by reversions could encode antigenic neopeptides [48]. This could suggest that therapeutic approaches that activate immune responses to these neopeptides could target reversion-mediated resistance. To this end, future work should establish whether neopeptide antigens are presented by class I MHC, and whether a robust and specific T-cell response can be induced against these neopeptides. If this proves to be the case, it may be possible to design vaccines based on candidate neopeptides predicted to occur upon reversion and to use these to prevent or delay *BRCA1/2* revertant tumour outgrowth.

### 4.2. Targeting PARPi resistance when caused by non-reversion-based mechanisms

Pre-clinical studies have suggested that PARPi resistance in *BRCA1* mutant cancers could emerge via loss of DNA end resection inhibitors (e.g. 53BP1, REV7, Shieldin) and the restoration of DNA resection (see earlier). Recent work suggests that while loss of these end resection inhibitors causes PARPi resistance, it also imparts upon tumour cells enhanced sensitivity to either ATR inhibitors [88], ionizing radiation [57], Pol*θ* inhibitors [89,90] or LIG3 inhibition [30]. To fully realise the potential of using these approaches to either delay or target PARPi once it has occurred, biomarkers that identify these non-reversion-based mechanisms of resistance are required, so that patients can receive an appropriate treatment (see earlier).

### 4.3. Using drug combination approaches to target PARPi resistance

One approach to improving the overall efficacy of cancer treatments is to use these in combination with other treatments. Historically, PARPi have generally been combined with other drugs that target DDR defects, including DNA damaging chemotherapies [91–95]. However, the clinical experience with such combinations has not been wholly positive, with dose-limiting toxicity being an issue [96–100]. What has been more profitable has been to combine PARPi with drugs that have different mechanism of action. When the drugs involved in combinations have different mechanisms of action, the possibility of resistance-mechanisms emerging that cause resistance to both agents (cross resistance) is minimized and thus the overall efficacy of treatment could be improved [101]. It is possible that this is the case with the approved bevacizumab/olaparib combination used in ovarian cancers, although some have suggested that this combination works because VEGF inhibition causes a HR defect [102–104].

Whether the combination of PARPi with additional agents that target other, mechanistically independent, drivers in cancer turn out to be effective remains to be seen. To this end, a series of clinical trials are currently underway where PARPi are used in combination with agents that target PI3 K/AKT (NCT04729387, NCT02208375, NCT04586335, NCT03586661, NCT02338622, NCT01623349), MEK1/2 (NCT03162627) signalling, and other pathways. For example, in metastatic, castration-resistant, prostate cancer (mCRPC), where PARPi are already approved for use in those with tumoural mutations in a series of BRCAness genes [69,105], androgen signalling inhibitors such as abiraterone acetate (a CYP17 inhibitor) and enzalutamide (an androgen receptor antagonist) are also part of the standard-of-care [106]. A phase I trial has already established the safety and tolerable dose of the PARPi niraparib when used in combination with abiraterone acetate plus prednisone (AAP) [107]. This combination is now being investigated further in a larger randomized placebo-controlled, phase 3 study in patients with mCRPC (MAGNITUDE; NCT03748641). Similarly, recent data from the PROpel Phase III trial indicate that the combination of olaparib plus abiraterone acetate deliver an improvement in radiographic progression-free survival (rPFS) as a first line treatment for men mCRPC, when compared to standard-of-care abiraterone [108]. Whether the drugs in this combination act synergistically or independently on different vulnerabilities in the same cancer remains to be seen. HR defects are relatively common in mCRPC as is the addiction to androgen signalling, suggesting that if the two drug types act independently, clinical benefit could be achieved as tumour clones with resistance mechanisms to one agent (such as reversion mutation in a HR gene) might still exhibit sensitivity to the second agent (targeting the androgen signalling addiction). Alternatively, it is possible that some synergistic interaction between the two drug classes contributes to the therapeutic effect; some studies have shown that using AR signalling inhibitors cause reduced expression of HR-associated genes, including *BRCA1*, *RAD54 L* and *RMI2* [109] and that loss of AR signalling reduces ATM signalling and MRE11 foci formation [110], effects that could be synthetic lethal with PARPi.

## 5. Concluding remarks

Over 50 years have passed since the first description of PARP1 function, 20 years since clinical trials using PARPi were initiated and close to eight years have passed since the first clinical approval of a PARPi [111–114]. Yet still, there is much to be discovered about PARPi that could refine how these drugs are used clinically. Some of these discoveries will no doubt come from pre-clinical work, but we also foresee a greater contribution to this field coming from ‘reverse translation’ where clinical observations made in people receiving PARPi as part of their cancer treatment tells us much about how these drugs work, and where we might better use these in the future. While we have summarized some of the key questions pertaining to refining the clinical use of PARPi, there are also other areas of research we have not covered. For example, how PARPi treatment influences the behaviour of patients' immune systems is very likely to have an impact on the overall clinical efficacy of these drugs [115,116] and no doubt further studies of those receiving PARPi will highlight how other bodily systems influence therapeutic responses.

## Data Availability

This article has no additional data.
